# Normative data for macular perimetry using the MP-3 microperimeter in healthy individuals

**DOI:** 10.5935/0004-2749.2021-0472

**Published:** 2023-03-08

**Authors:** Taurino dos Santos Rodrigues Neto, Epitácio Dias da Silva Neto, Alex Haruo Higashi, Bianca Partezani Megnis, Maria Aparecida Onuki Haddad, Mário Luiz Ribeiro Monteiro, Leandro Cabral Zacharias

**Affiliations:** 1 Division of Ophthalmology, Laboratory for Investigation in Ophthalmology (LIM-33), Faculdade de Medicina, Universidade de São Paulo, São Paulo, SP, Brazil

**Keywords:** Visual fields, Visual field tests, Retina, Microperimetry, Age, Campos visual, Testes de campo visual, Retina, Microperímetro, Idade

## Abstract

**Purpose:**

Microperimetry has been used for several years as a form of visual function testing in patients with retinal diseases. Normal microperimetry values obtained with microperimeter MP-3 have not yet been fully published, and baseline values for topographic macular sensitivity and correlations with age and sex are needed to establish degrees of impairment. This study aimed to determine values for light sensitivity thresholds and fixation stability using the MP-3 in healthy individuals.

**Methods:**

Thirty-seven healthy volunteers (age, 28-68 years), underwent full-threshold microperimetry using a 4-2 (fast) staircase strategy with the standard Goldmann III stimulus size and 68 test points positioned identically to those in the Humphrey Field Analyzer 10-2 test grid. The fixation stability was simultaneously recorded during the microperimetry test. The relationship between global sensitivity and age was calculated using linear regression analysis.

**Results:**

Microperimetry was performed on 37 participants (74 eyes). The global mean sensitivity was 29.01 ± 1.44 (range, 26-31) dB. The mean central sensitivity at 2° measured by the MP-3 was 28.5 ± 1.77 dB in the right eye (OD) and 28.75 ± 1.98 dB in the left eye (OS). The total median fixation stability values within 2° and 4° were 80% and 96%, respectively. The linear regression analysis also revealed an age-related global sensitivity decline per year of -0.051 dB ± 0.018 (OD) and -0.078 dB ± 0.021 (OS).

**Conclusions:**

Microperimetry performed with the MP-3 allows for an automatic, accurate, and topography-specific examination of retinal sensitivity thresholds. The results of this study provide a normal and age-matched database of MP-3 microperimetry.

## Abstract (pt)

**Objetivos:**

A microperimetria tem sido usada há vários anos como uma forma de teste de função visual em pacientes com doenças da retina. Os valores normais de microperimetria obtidos com MP-3 ainda não foram totalmente publicados e os valores basais para sensibilidade macular topográfica e correlações com idade e sexo são necessários para estabelecer graus de comprometimento. O objetivo do trabalho é determinar valores para limiares de sensibilidade à luz e estabilidade de fixação usando o MP-3 em indivíduos normais.

**Métodos:**

Trinta e sete voluntários saudáveis (idade: 28-68 anos), submetidos à microperimetria de limiar total usando uma estratégia de escada 4-2 (rápida) com o tamanho de estímulo padrão Goldmann III e 68 pontos de teste posicionados de forma idêntica aos do Humphrey Field Analyzer 10-2 grade de teste. A estabilidade da fixação foi registrada simultaneamente durante o teste de microperimetria. A relação entre a sensibilidade global e a idade foi calculada por meio de análise de regressão linear.

**Resultados:**

A microperimetria foi realizada em 37 indivíduos (74 olhos). A sensibilidade média global foi de 29,01 ± 1,44 dB, intervalo: 26-31 dB. A mediana da sensibilidade central a 2° medida pelo MP-3 foi de 28,5 ± 1,77 dB (ER) e 28,75 ± 1,98 dB (OE). Os valores médios totais de estabilidade da fixação em 2° e 4° foram 80% e 96%, respectivamente. A análise de regressão linear também revelou um declínio de sensibilidade global relacionado à idade por ano de -0,051 dB ± 0,018 (ER) e -0,078 dB ± 0,021 (LE).

**Conclusões:**

A microperimetria realizada com o MP-3 permite um exame automático, preciso e específico da topografia dos limiares de sensibilidade da retina. Os resultados deste estudo fornecem um banco de dados normal e de idade correspondente da microperimetria MP-3.

## INTRODUCTION

Microperimetry is a promising functional test for the screening and follow-up of macular diseases^(1)^. It can estimate retinal sensitivity for a certain macular point, with its accuracy based on a fixation area on the retina, which is the fundamental difference between standard automated perimetry (SAP) and microperimetry when assessing retinal sensitivity. In SAP, stimuli are projected on a screen in front of the eye, and acceptable fixation maintenance during the test is relative to the size of the natural blind spot. In microperimetry, stimuli are projected directly onto the retina, and accurate test-retest of the same retinal point is monitored by eye-tracking technology, thus minimizing the effect of poor fixation and providing a fundus image registered over the retinal sensitivity measurements for the clinician to review^(2)^.

In the past few years, microperimetry quickly became a popular and reproducible method to evaluate visual function^(3)^. The Nidek MP-1 was one of the first microparameters to become commercially available. A programmable projection system allows the delivery of modulated stimuli in the macular area. The MP-1 has been reported to be clinically useful in various central retinal pathologies^(4-6)^.

A new microperimeter with improved fundus image tracking (MP-3, Nidek Co., Ltd., Japan) became available recently. The system comprises a nonmydriatic fundus camera with a 45° field of view. During the measurement, an infrared image is used for motion capture. In contrast to previous devices, its tracking frame rate is 30 Hz, which means that proper fixation and the correct position of the stimulation grid are evaluated 30 times per second. The eye tracker compensates for ocular movements during testing and ensures that point-to--point correspondence exists between the stimulus and the measured retinal location during the test^(7)^. These functions allow for easy follow-up and reduce variations between examiners, resulting in well-aligned follow-up examinations and greater inter-test reproducibility.

Previous studies with the MP-1 have established age--matched normative data^(8,9)^. However, normative data and reference values according to age for the retinal light sensitivities for MP-3 have not been established. These are of paramount importance to draw meaningful conclusions in the pathological setting. This study aimed to evaluate light sensitivity thresholds and fixation stability using the MP-3 in a healthy Brazilian population.

## METHODS

### Study participants

This cross-sectional, observational, and descriptive study followed the precepts of the Declaration of Helsinki (1996), Nuremberg Code (1947), Research Norms Involving Humans chosen in resolution 196/96 of the National Health Council, and our Institutional Review Board Ethics Committee. All participants provided written informed consent before study enrollment.

The inclusion criteria were as follows: consenting adults aged ≥18 years, healthy eyes, and clear ocular media. The exclusion criteria were as follows: diabetes mellitus, serious chronic systemic diseases, previous brain surgery, ocular surgery, and ocular diseases that might affect the retina, choroid, or optic nerve (such as retinopathies, uveitis, optic neuropathies, or abnormalities), high myopia (axial length > 26.5 or spherical refraction <-6 diopters), high hyperopia (spherical refraction >+6 diopters), cylinder refraction >±3 diopters, and intraocular pressure >21 mmHg.

### Ophthalmologic examination and MP-3 perimeter acquisition

Each participant received a comprehensive ophthalmology examination including manifest refraction, slit--lamp examination, axial length measurement, intrao-cular pressure measurement, and dilated fundus examination. Microperimetry examination with the MP-3 perimeter was performed in both eyes of all patients. Patients’ eyes were dilated with one drop of tropicamide 1% 30 min before the examination. For fixation, the target was a red cross, 1° in diameter. To determine the retinal light sensitivity threshold, a red ring fixation target, 1° in diameter, was used on a white, monochromatic background at 31.4 apostilb (asb). A Goldman III stimulus size was chosen, with a 200-ms projection time. The maximum luminance of the MP-3 is 10 000 asb, and the stimulus dynamic range is between 0 and 34 dB. A personalized grid of 68 test points was positioned identically to the observed target in the Humphrey field analyzer 10-2 test grid. The 4-2 (fast) scheduling method was used with an automatic eye tracker. Briefly, in this strategy, a stimulus of greater than expected intensity is presented; if seen, the intensity is reduced in steps of 4 dB until it is no longer observed, increasing the stimulus again at 2 dB intervals, until it is seen again. Self-tracking and auto-alignment functions ensured accurate measurements. A flash color fundus photograph, with a resolution of 1388 × 1038 pixels and covering 45° of the field, was taken at the end of the examination. This allows the visual function to be superposed and therefore compared with retinal structures.

### MP-3 perimeter analysis

**Figure 1:**
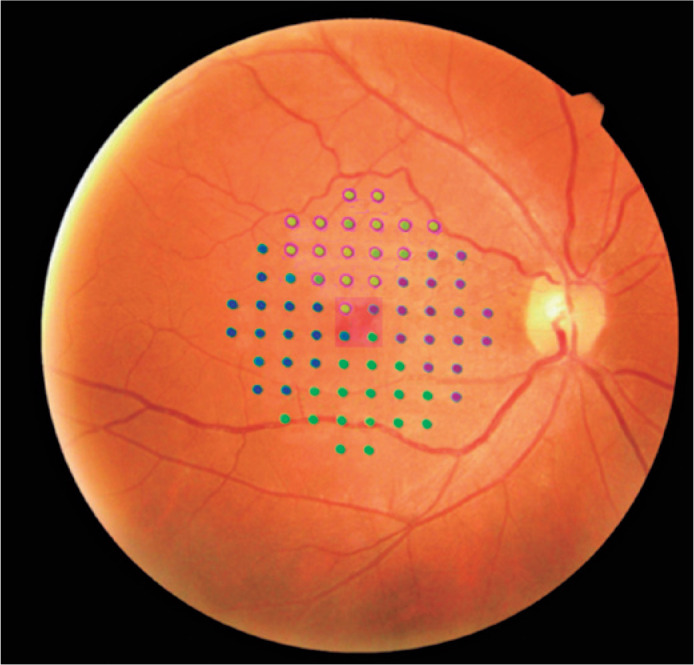
Retinal points evaluated with the MP-3 microperimeter (Nidek Co., Ltd., Aichi, Japan). This grid consisted of 68 stimuli centered on the fovea: the central 2° region consisted of four central points (red square) and divided by each sector (superior, green/purple; nasal, purple; temporal, blue; and inferior, green).

The mean macular sensitivity at 2° and the median sensitivity for each sector (superior, nasal, temporal, and inferior) were calculated (Figure 1). The global mean sensitivity (arithmetic average) of the 68 points studied and the retinal mean sensitivity in every 68 points of both eyes were measured. It also analyzes the patient’s fixation stability and determines the preferred retinal locus. The instrument automatically calculates and displays the fixation stability.

The fixation stability analysis was classified based on a degree circle that had the center of the centroid of all fixation points. The recorded fixation patterns were divided into three groups, as follows: stable, >75% of the fixation points are inside a 2° diameter circle; relatively unstable, >75% of the fixation points are inside a 4° diameter circle and <75% are inside the 2° diameter circle; or as unstable, <75% of the fixation points are inside the 4° diameter circle.

More accurate estimates of the fixation stability were quantified using the bivariate contour ellipse area (BCEA), which is a mathematical model that can describe irregularly shaped sets of points. The BCEA method calculates the area and orientation of an ellipse encompassing a given proportion of the fixation points dataset, where lower BCEA values indicate better fixation stability. However, it does not have widely accepted cutoffs to distinguish stable from unstable fixation. The fixation stability using BCEA is based on the standardized fixation points that eliminate the extreme outlier coordinates beyond ±3 standard deviation (SD) (68.2%, 95.4%, and 99.6%). The area values observed in the 1st SD ellipse (68.2%) were exported.

### Data analysis and statistics

Continuous variables were expressed as mean and SD or median and interquartile range (IQR). Normality was analyzed by the Shapiro-Wilk test. Student’s t-test was used for normally distributed continuous variables, and the Mann-Whitney test for asymmetrical continuous variables. Spearman’s correlation coefficient (R) and linear regression analysis were used. A p-value of <0.05 was considered statistically significant. Data were analyzed using the SPSS Statistical Package for Windows, version 17 (SPSS, Inc., Chicago, IL, USA).

## RESULTS

A total of 37 healthy volunteers were enrolled in this study between January and March 2020. The average age was 41 (28-68) years, the average axial length was 23.19 (21.1-26.06) mm on the right eye (OD) and 23.05 (21.3-25.9) mm on the left eye (OS). The average IOP was 15 mmHg. All tests were reliable.

### Macular sensitivity

**Figure 2:**
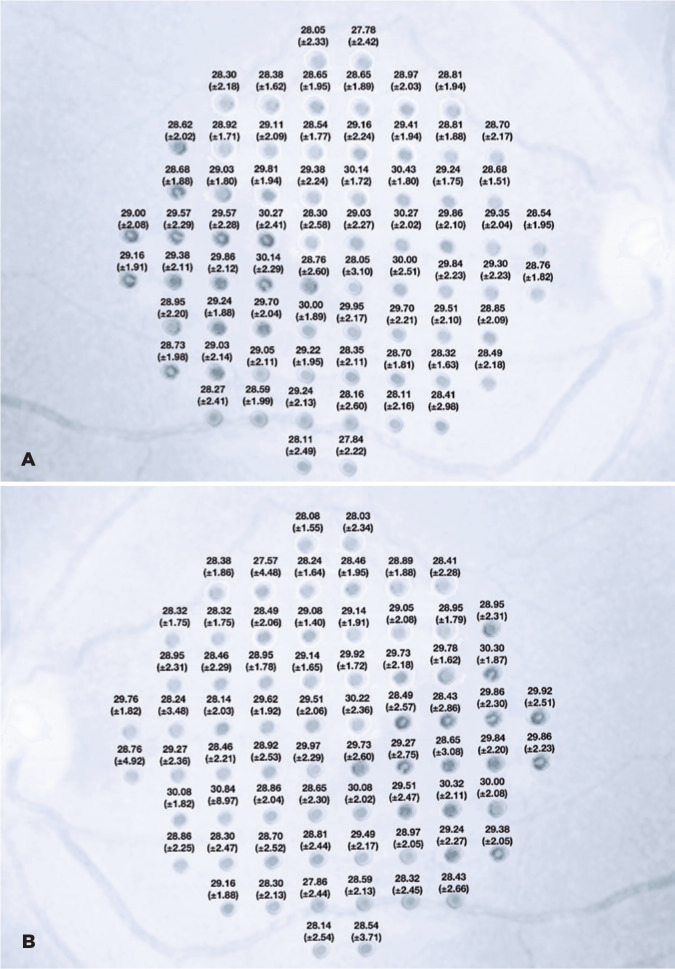
Retinal mean sensitivity in each 68 points of the right eye (A) and the left eye (B) evaluated with the MP-3 microperimeter (Nidek Co., Ltd., Aichi, Japan).

The global mean sensitivities over all the points on the retina were 29.01 ± 1.44 dB, 29.09 ± 1.26 dB (OD), and 28.93 ± 1.61 dB (OS). The retinal mean sensitivity in every 68 points of both eyes is described in figure 2. The median sensitivities on each sector were as follows: inferior, 492 dB; superior, 488 dB; nasal, 495 dB; and temporal, 506 dB. The pairwise comparison demonstrated a difference between the superior and temporal sectors (p=0.021), without difference among other sectors (Table 1).

**Table 1:** Comparison of the median sector sensitivity measured by the MP-3 (Nidek Co., Ltd., Aichi, Japan).

	Inferior	Superior	Nasal	Temporal
N	74	74	74	74
Median	492	488	495	506
IQR	473-512	473-508	481-518	485-518
Minimum	401	425	444	381
Maximum	533	528	539	532

IQR= interquartile range; N= number.Kruskal-Wallis test, p=0.022; Dwass-Steel- Critchlow-Fligner pairwise comparison superior vs. temporal; p=0.021. Without differences with the pairwise comparisons of other sectors.

**Figure 3:**
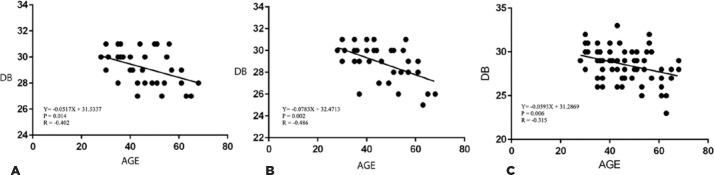
Linear regression analysis between the global mean sensitivity and age as measured by the MP-3 in the right eye (A) and the left eye (B). (C) Relationship between the central mean sensitivity and age as measured by the MP-3 (Nidek Co., Ltd., Aichi, Japan).

**Figure 4:**
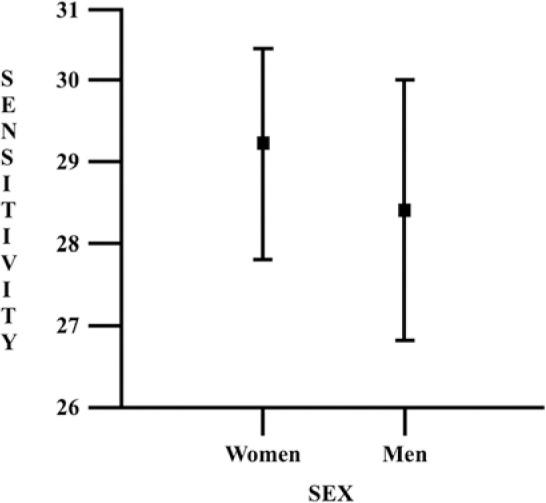
Boxplot chart illustrates the mean global sensitivity values by sex determined with the MP-3 microperimeter (Nidek Co., Ltd, Aichi, Japan).

The mean central sensitivities at 2° measured by the MP-3 were 28.40 ± 1.86 dB, 28.32 ± 1.77 dB (OD), and 28.48 ± 1.98 dB (OS). No statistically significant difference was found between the global and central mean sensitivities (p=0.10). A linear decline in macular sensitivity with age was found, which was measured by the MP-3. The linear regression analysis revealed -0.051 dB ± 0.018 (OD) and -0.078 dB ± 0.021 (OS) per year age-related decline in global sensitivity (Figures 3A and 3B). The linear regression for central sensitivity revealed -0.059 ± 0.019 dB of age-related decline (Figure 3C). No statistically significant differences were found between the global mean sensitivities for sex (p=0.11) or eye (p=0.43). The sensitivity by sex is presented in Figure 4. The median fixation stability on 2° and 4° circles were 80% and 96%, respectively, and the BCEA measured by the MP-3 in the 1st SD ellipse (68.2%) was 2.25°^(2)^. The fixation by sex is presented in table 2.

**Table 2:** Sex comparison of the mean global sensitivity, median fixation stability on 2° and 4° degree circle and bivariate contour ellipse area measured by the MP-3 (Nidek Co., Ltd., Aichi, Japan)

Sex	Global sensitivity (mean)	Fixation 2° (median) %	Fixation 4° (median) %	BCEA (1SD-AREA) (median)
Male (14 eyes)	28.46 dB	62.50	93.50	3.2°^2^
Female (60 eyes)	29.18 dB	83.50	97	2.0°^2^
p-value	0.11	<0.0001	0.0240	0.015
Mann-Whitney rank-sum test.				

## DISCUSSION

The use of microperimetry in the diagnosis and assessment of macular disease is a promising tool to enhance the understanding of macular disease and the assessment of future and existing treatments. Macular examination with microperimetry is an ideal tool to assess retinal sensitivity and fixation behavior in patients with macular diseases. Measuring macular function has become common in the assessment of natural history and treatment outcomes in macular disease because it enables exact correlation analysis between macular pathologies and corresponding functional abnormalities. In recent publications, the MP-3 is clinically useful in several central retinal pathologies^(7,10)^. However, normative data and reference values according to age for retinal light sensitivities have not been established. These are of paramount importance to draw meaningful conclusions in a pathological setting.

In a study comparing differential retinal sensitivity measurements obtained with MP-3 and the CenterVue Macular Integrity Assessment (MAIA) microperimeters among healthy participants, testing 37-stimuli grid overlying the central 10°, the mean retinal sensitivity (dB) measured by the MP-3 was 25.02 ± 1.06 (range, 20.90-26.70) dB^(11)^. In the present study, we tested a 68-stimuli grid overlying the central 10° and found that the mean retinal sensitivity was 29.09 ± 1.44 (range, 26-31) dB. A possible explanation for the difference in retinal sensitivity could be the larger number of points in our study or the distribution of these points. In our study, the 68 points were positioned identically to the observed object in the Humphrey field analyzer 10-2 test grid, and in the other study, the 37 points were standardized as a stimulus grid consisting of a single central foveal response and three concentric rings of the retinal loci at 2°, 6° and 10° from the central point.

Stimulus location and age are critical parameters that influence differential light threshold values in healthy participants. Therefore, these parameters should be considered when interpreting the results in both healthy and pathologic eyes. Some pathogenic factors of the preretinal origin, such as a reduction of pupil size, ocular media opacities, and neural loss, have been proposed to account for this age-related reduction in retinal sensitivity. Studies have reported age-related linear regression in MP1. The mean retinal sensitivity (dB) measured by the MP-1 was 19.3 ± 0.9 (range, 15.8-20) dB, and the linear regression analysis revealed a -0.019 dB per year age-related decline in the central mean macular sensitivity at 2°^(8,9)^. However, it is not well established in MP-3 yet. In the linear regression analysis, the results were -0.059 ± 0.019 dB in the central 2° and -0.051 ± 0.018 dB (OD) and -0.078 ± 0.021 dB (OS) per year age-related decline in global mean sensitivity. In previous MP1 studies, light sensitivity linearly reduced with age in healthy participants. Normal threshold values obtained with the MP-3 microperimeter cannot be currently compared with those obtained with the MP-1 because the intensity of the stimuli in the machine ranged from 0 to 20 dB, which is lower than the MP-3 stimulus dynamic range, between 0 and 34 dB^(9,12,13)^.

The limitations of this study include the relatively small number of participants, having only one measurement per eye, and only eyes without ocular pathologies were examined. Larger comprehensive studies with the MP-3 are recommended to corroborate our findings and address normal interpersonal variations (between participants of the same age and sex) and intrapersonal fluctuation with MP-3, as it was previously described with MAIA microperimeter^(14)^.

In conclusion, microperimetry performed with the MP-3 allows for an automatic, accurate, repeatable, and topography-specific examination of retinal sensitivity thresholds. Knowledge of normal threshold values is critical, especially when shallow defects are present. The results of this study provide a normal and age-matched database of MP-3 microperimetry.
